# Favorable impact of long-term exercise on disease symptoms in pediatric patients with inflammatory bowel disease

**DOI:** 10.1186/s12887-019-1680-7

**Published:** 2019-08-27

**Authors:** Corinne Legeret, Laura Mählmann, Markus Gerber, Nadeem Kalak, Henrik Köhler, Edith Holsboer-Trachsler, Serge Brand, Raoul Furlano

**Affiliations:** 1 Children’s University Hospital of Basel, Spitalstrasse 33, 4056 Basel, Switzerland; 2 Children’s Hospital of Aarau, Aarau, Switzerland; 3 Psychiatric Clinics of the University of Basel, Centre for Affective, Stress and Sleep Disorders, University of Basel, Basel, Switzerland; 4 Department of Sport, Exercise and Health, Sport Science Section, University of Basel, Basel, Switzerland; 5 Psychiatry Department, Substance Abuse Prevention and Sleep Disorders Research Center, Kermanshah University of Medical Sciences (KUMS), Kermanshah, Iran

**Keywords:** Physical activity, Exergaming, Inflammatory bowel disease, Pediatrics, Inflammation

## Abstract

**Background:**

Evidence is growing that both short- and long-term physical exercise have the potential to positively impact on the physiological system related to inflammatory indices, though, such patterns are unknown for pediatric patients with Inflammatory Bowel Disease (IBD). The aim of the present intervention study was to investigate the influence of a single bout and chronic moderate-intensity exercise on IBD-related inflammatory indices and exercise capacity among pediatric individuals with IBD and healthy controls.

**Method:**

Twenty-one pediatric patients with IBD, split into a “remission-group” (IBD-RE; *n* = 14) and an “active disease group” (IBD-AD; *n* = 7), were compared to 23 age matched healthy controls (HC). All participants completed a single bout of exercise at baseline and an 8-week exercise intervention. Before and after the single bout of exercise IBD-related inflammatory indices (erythrocyte sedimentation rate (ESR), albumin, C-reactive protein (CRP), cortisol, hemoglobin, hematocrit, thrombocytes and leukocytes) were assessed.

**Results:**

At baseline, after a single bout of exercise, inflammation (albumin, hemoglobin, erythrocytes, hematocrit and leukocytes) increased in all three groups IBD-AD, IBD-RE and HC. CRP and thrombocytes were only elevated in IBD-AD and IBD-RE, compared to HC. After a longer-term exercise intervention, ESR, CRP and thrombocytes significantly decreased in all groups. The longer-term exercise intervention did not decrease acute immunopathologic responses after a single bout of exercise, compared to baseline.

**Conclusion:**

Whereas a single bout of exercise increases albumin, erythrocytes and leukocytes, longer-term moderate-intensity exercise reduced inflammatory markers in pediatric patients with IBD. Children and teenagers with IBD should be encouraged to engage in regular moderate-intensity exercise activities, as such activities may contribute to inflammation suppression and improved disease management.

## Background

Inflammatory bowel disease (IBD) is a chronic inflammation affecting the gastrointestinal tract. It is commonly divided into two forms: Crohn’s disease (CD) and Ulcerative colitis (UC) [1]. Whilst UC affects the colon, CD can promote an inflammation in any part of the digestive system and for both an increase in prevalence and incidence can be observed [2]. Symptoms can vary from chronic anemia, abdominal pain, failure to thrive, bloody diarrhea to toxic megacolon.

The current standard of care for pediatric patients with IBD includes a combination of pharmacotherapy, surgical intervention, and/or enteral nutrition [3]. Physical activity has frequently been suggested as adjuvant therapy option in the prevention and treatment of a variety of chronic and inflammatory diseases [4–6]. In addition to the favorable effect on physical, psychological/social and cognitive dimensions [7, 8] physical activity has a beneficial impact on bone mineral density, muscle mass, functional capacity [7] and also seem to have immune modulating potential [9–11].

Concerning long-term physical activity interventions in individuals with IBD, several studies [15–22] and four reviews [7, 12–14] have been published. A limited number of studies indicate a beneficial effect of physical activity on the quality of life [15, 18], psychological and physical aspects [17] and physiological symptoms [20] for individuals with IBD. A randomized control trial over a 12-week intervention reported improvements in constipation symptoms [20]. Unfortunately, the effectiveness of single or longer-term exercise has not been well described [15–22], the processes are poorly understood and limited to the adult population [12, 23].

With this in mind, the aim of the current study was to test the immunopathologic response to a single bout of exercise after a longer-term intervention in children with IBD.

Our research question was, whether the immunopathologic response to a single bout of exercise would be reduced after a longer-term intervention. We also formulated two hypotheses: First, we expected higher inflammatory indices after a single bout of moderate-intensity exercise among pediatric patients with IBD and healthy controls, as in other studies [24, 25] higher pro-inflammatory cytokines in the same setting were described. Second, after consulting the literature [12–14] we expected that a longer-term moderate-intensity physical activity intervention would favorably impact the inflammatory blood markers in pediatric patients with IBD and healthy controls.

## Methods

As previously described [26] in this case-control 8-week longer-term physical activity exercise intervention study, patients with IBD were recruited from the University Children’s Hospital Basel and the children’s hospital Aarau (Switzerland). Exclusion criteria were the presence of another chronic disease and/or intake of regular medication, other than to treat IBD. Patients, who were interested in participating were asked to bring an aged matched healthy friend (chronic disease and regular intake of medication were exclusion criteria) for the healthy control group (HC). All participants were invited to the hospital to perform a single bout of exercise and an assessment immediately before and after the physical intervention. All participants were fully informed about the aims of the present study, and the voluntary and confidential basis of their participation. Thereafter, written informed consent was signed by participants and their legal guardians. All data were collected by two trained research assistants and supervised by a medical doctor.

### Procedures

Before and after a single bout (6-min walking test) and long-term exercises (8 weeks), IBD-related inflammatory indices (erythrocyte sedimentation rate (ESR), albumin, C-reactive protein (CRP), cortisol, hemoglobin, hematocrit, thrombocytes and leukocytes) were assessed, afterwards anthropometric measures were taken.

The present study was approved by the local ethical committee (Ethics committee north-west of Switzerland, EKNZ: 2014:220). Furthermore, the study was conducted in accordance to the ethical principles laid down in the Declaration of Helsinki and its later amendments (Trial registration number: NCT02264275).

Individuals with IBD were subdivided according to the activity scores of their disease (PUCAI [Paediatric ulcerative colitis activity index] and PCDAI [Paediatric Crohn’s disease activity index]) [27]. Clinical remission was defined as PUCAI/PCDAI score of 10 and below. Medical treatment was not changed during the course of the study (see Table 3).

## Tools

### Anthropometric dimensions

Height and waist circumference (measured 4 cm above the navel using a standard anthropometry tape) were objectively measured to the nearest 0.1 cm. Weight was taken in kg and BMI (kg/m^2^) was calculated and compared to reference values established by Taylor et al. [28] and to the WHO international growth references [29].

### Laboratory assessments

Blood samples were obtained using the finger prick technique to measure inflammatory indices such as CRP [g/dl], thrombocyte [g/dl] and leukocyte [g/dl] count, as well as hemoglobin (Hb) [g/dl], albumin, cortisol and erythrocyte sedimentation rate [ESR].

## Exercise interventions

### Single bout of exercise

To assess the effects of a single bout of exercise, a self-paced, submaximal 6-min walking test (6MWT) was applied as part of the baseline and follow-up data assessment. The 6MWT measures the maximal walking distance of the participants over 6 min [30]. This was chosen as it has been validated as suitable for pediatric populations with chronic diseases [31].

### Longer-term physical intervention

As described previously [26], the longer-term physical intervention incorporated an exergame or active video gameplay. Exergames were selected since they are easily accessible with adjustable levels of intensity to minimalize perceived barriers, perceived threads and as an intervention to facilitate action taking in a safe environment [32–37]. Exergames can elevate energy expenditure to a moderate or vigorous intensity, metabolically equivalent to a three mile per hour treadmill pace [32]. Furthermore, gamification techniques and mechanisms enable to achieve goals, encourage players through rewards and points, while offering intrinsic motivation in the form of fun.

After the review of various exergames and their intensity levels, we decided to use the Just Dance Kids® and Sports-Resort® exergame for Nintendo Wii® in the present study. Just Dance Kids® is a motion-based dancing game offering the player a collection of songs with accompanied dance choreography. During the songs, players mirror the dance performance and comments displayed on the screen and are awarded for their accuracy. The Nintendo Wii Sports-Resorts® game allows children to participate in a sports game in a virtual world. Players can choose between sward play, wakeboarding, frisbee, archery, basketball, table tennis, golf, bowling, canoeing and cycling. Players can either compete against the video game or can play against a second player.

Children and adolescents were offered to choose one of the games and were encouraged to use the exergames for 30 min, 5 days a week for a total of 8 weeks. Participant’s parents were asked to monitor the intervention frequency in a paper diary to keep track of intervention adherence over the entire intervention period.

### Statistical analyses

Anthropometric measures, blood values and physical activity outcomes by group (IBD-AD, IBD-RE and HC) and time (baseline vs post-intervention) were tested with analysis of variance (ANOVA) for repeated measures. Post-hoc tests after Bonferroni-Holm corrections were performed to examine differences between and within the three specific groups. Given the deviation of sphericity, Greenhouse-Geisser adjusted the degrees of freedom. The slope of increasing blood markers between pre and post single bout of exercise was calculated with [(T0 x T1) / 2] and the area under the curve (AUC), representing total inflammation, was calculated with [((T0 x T1) / 2) × 6]. Significance level was set at alpha *p* < .05. All statistics were performed with SPSS® 25.0 (IBM Corporation, Armonk NY, USA) for Windows®.

## Results

From over fifty patients with IBD, twenty-three agreed to participate. After 2 drop-outs the study was finished with twenty-one pediatric patients with IBD (mean age: 13.35 years; females *n* = 10 [43.5%]): Seven participants (females *n* = 2) were in an active disease state (IBD-AD) and 14 (females *n* = 8) were in remission (IBD-RE) during the study period. The mean age of the healthy control group (HC) was 12.38 years (females *n* = 15 [62.5%]). For further details regarding the characteristics we refer to Table 1.

**Table 1:** Sample Characteristics

	IBD-AD	IBD-RE	HC
	*(n = 7)*	*(n = 14)*	*(n = 23)*
Age in years	12.78 ± 3.25	13.64 ± 2.96	12.38 ± 3.23
Female	2 (28.6%)	8 (57.14%)	15 (62.5%)
IBD			
Ulcerative colitis	4	5	
Crohn’s Disease	3	8	
Indeterminate colitis		1	
Time since diagnosis	111.54 ± 83.35	124.74 ± 88.19	
Height (cm)	153.48 ± 17.53	152.69 ± 12.02	152.26 ± 18.0
Weight (kg)	45.28 ± 15.85	42.79 ± 12.83	44.37 ± 15.75
Weight to height (%)	44.08 ± 3.07	42.94 ± 2.13	42.77 ± 3.49
BMI	18.6 ± 3.37	17.91 ± 2.78	18.36 ± 3.16

*Notes: N* = 44, IBD-AD = IBD in an active state of the disease, IBD-RE = IBD in remission, HC = Healthy Control; all data presented in mean ± standard deviation

### Immunopathologic response after a single bout of exercise at baseline

Table 2 provides an overview of the descriptive and inferential statistics for blood markers before and after a single exercise bout (6MWT). All results are presented separately by time (before vs after single bout), groups (IBD-AD vs. IBD-RE vs. HC), and interaction (group x time).

**Table 2:** Results

	IBD-AD		IBD-RE		HC		*Time*			*Group*			*Group x Time*		
	*(n = 7)*		*(n = 14)*		*(n = 23)*										
	*Pretest (M)*	*Posttest (M)*	*Pretest (M)*	*Posttest (M)*	*Pretest (M)*	*Posttest (M)*	*F*	*p*	*ηp* ^*2*^	*F*	*p*	*ηp* ^*2*^	*F*	*p*	*ηp* ^*2*^
Inflammation before and after a single bout of exercise															
ESR	11.83 ± 8.06	9.67 ± 7.4	11.83 ± 13.17	8.5 ± 10.11	6.05 ± 6.57	7.59 ± 9.42	0.61	.44	0.02	0.97	.39	0.05	1.19	.32	0.06
Albumin	19.36 ± 17.79	20.04 ± 18.57	20.37 ± 18.77	21.08 ± 19.5	21.49 ± 18.63	22.36 ± 19.44	17.80	.00	0.30	0.05	.96	0.00	0.13	.88	0.01
CRP	4.44 ± 5.57	4.52 ± 5.7	3.02 ± 4.56	3.02 ± 4.51	0.67 ± 0.84	0.7 ± 0.84	1.91	.18	0.05	4.10	.02	0.18	0.85	.43	0.04
Cortisol	289.75 ± 159.82	285.63 ± 191.25	248.31 ± 138.53	242 ± 146.79	203.83 ± 109.8	208.35 ± 104.4	0.06	.81	0.00	1.26	.29	0.06	0.23	.80	0.01
Hemoglobin	126 ± 19.57	129.88 ± 21.03	135.31 ± 15.63	135.77 ± 15.88	135.91 ± 12.05	136.78 ± 13.57	8.05	.01	0.16	0.96	.39	0.04	2.42	.10	0.11
Erytrozytes	4.78 ± 0.28	4.93 ± 0.25	4.87 ± 0.51	4.90 ± 0.52	4.87 ± 0.35	4.89 ± 0.37	11.86	.00	0.22	0.01	.99	0.00	3.23	.05	0.14
Hematocrit	37.06 ± 4.13	38.29 ± 4.2	38.17 ± 3.83	38.62 ± 3.98	38.7 ± 3.11	39.02 ± 3.41	17.71	.00	0.30	0.33	.72	0.02	2.71	.08	0.12
Thrombocytes	416.5 ± 181.38	402.13 ± 102.78	310.46 ± 60.80	334.85 ± 68.85	270.87 ± 45.59	293.52 ± 55.62	1.88	.18	0.04	8.38	.00	0.29	2.01	.15	0.09
Leukocytes	9.5 ± 1.93	11.05 ± 2.2	9.28 ± 2.71	10.75 ± 3.11	8.23 ± 1.26	9.46 ± 1.95	38.54	.00	0.48	2.14	.13	0.09	0.21	.81	0.01
Inflammation before and after 8-week ET intervention															
ESR	24.5 ± 26.33	11.5 ± 16.56	11.83 ± 13.17	6.33 ± 5.85	6.24 ± 6.66	7.19 ± 7.77	8.69	.01	0.19	3.02	.06	0.14	4.21	.02	0.19
Albumin	24.83 ± 17.31	28.42 ± 19.24	20.37 ± 18.77	23.28 ± 18.73	23.14 ± 18.69	28.58 ± 17.99	2.47	.12	0.06	0.27	.76	0.01	0.14	.87	0.01
CRP	4.44 ± 5.57	0.9 ± 0.85	3.02 ± 4.56	0.63 ± 0.86	0.72 ± 0.87	0.92 ± 1.56	10.40	.00	0.23	2.31	.11	0.12	4.47	.02	0.20
Cortisol	214.67 ± 85.67	161.83 ± 79.75	248.31 ± 138.53	227.54 ± 111.15	209.05 ± 109.93	222.33 ± 99.53	0.92	.34	0.02	0.61	.55	0.03	0.88	.42	0.05
Hemoglobin	133.33 ± 11.55	136.5 ± 9.71	135.31 ± 15.63	135.54 ± 13.85	135.5 ± 12.16	133.68 ± 12.04	0.23	.64	0.01	0.02	.98	0.00	1.66	.20	0.08
Erytrozyten	4.78 ± 0.33	4.86 ± 0.42	4.87 ± 0.51	4.87 ± 0.44	4.85 ± 0.35	4.77 ± 0.25	0.03	.85	0.00	0.05	.95	0.00	0.80	.46	0.04

*IBD-AD* IBD in an active state of the disease, *IBD-RE* IBD in remission, *HC* Healthy Control, *BSG* Blood Sedimentation Grade, *CRP* C-Reactive Protein

*Notes. N* = 44; degrees of freedom always = 2, 41; p < .05 statistically significant; all data presented in mean (M) ± standard deviation; effect sizes: small (s) = .01 ≤ ηp^2^ ≤ .059, medium (m) = .06 ≤ ηp^2^ ≤ .139, or large (l) = ηp^2^ ≥ .14

Albumin (*p = .*00), hemoglobin (*p = .*01), erythrocytes (*p = .*00), hematocrit (*p = .*00) and leukocytes (*p = .*00) increased in all three groups (IBD-AD, IBD-RE and HC). No significant time effects were observed for ESR, CRP, cortisol and thrombocytes.

CRP-values were significantly higher in the IBD-AD and IBD-RE groups as compared to the HC group (*p = .*02). Furthermore, thrombocytes were highest in the IBD-AD group, followed by the IBD-RE group, and lowest in HC (*p = .*00). No significant group effects were found for BSG, albumin, cortisol, hemoglobin, erythrocytes, hematocrit and leukocytes.

Concerning interactions (time x group), no significant results were observed for any of the blood measures, namely for ESR, albumin, CRP, cortisol, hemoglobin, erythrocytes, hematocrit, thrombocytes and leukocytes.

### Immunopathologic response after longer-term intervention

Table 2 also provides an overview of the descriptive and inferential statistics for blood markers before and after the 8-week physical intervention.

Over time, ESR (*p = .*01), CRP (*p = .*00) and thrombocytes (*p = .*02) significantly decreased. No significant time effects were observed for albumin, cortisol, hemoglobin, erythrocytes, hematocrit and leukocytes.

Comparing groups, no significant chances were found for ESR, albumin, CRP, cortisol, hemoglobin, erythrocytes, hematocrit, thrombocytes and leukocytes.

Concerning interactions (time x group), significant effects were observed for ESR (*p = .*02), CRP (*p = .*02) and thrombocytes (*p < .*001). Post-hoc analyses with Bonferroni-Holm corrections for *p*-values revealed decreased values over time in IBD-AD and IBD-RE, while values for HC increased. Moreover, no significant time effects were detected for ESR, albumin, cortisol, hemoglobin, erythrocytes, hematocrit and leukocytes. There was no statistically significant change in clinical disease activity indices before and after the longer-term intervention, during which the treatment of the IBD was not changed (Table 3).

**Table 3:** Characteristics and medication of IBD patients

Patient	Age in years	Diagnosis	Medication	PUCAI/PCDAI before 8-week intervention	PUCAI/PCDAI after 8-week intervention
1	14.16	Crohn’s disease	Infliximab 5 mg/kg 6-weekly and Azathioprine 100 mg/ day	25	15
2	17.08	Crohn’s disease	Infliximab 5 mg/kg 6-weekly	30	25
3	11.75	Ulcerative colitis	Azathioprine 100 mg/day	0	0
4	17.16	Crohn’s disease	Ustekinumab 45 mg s.c. 8-weekly	5	5
5	13.41	Ulcerative colitis	Golimumab 50 mg s.c. every 4-weekly	0	5
6	14.16	Crohn’s disease	Mesalazine 2 g/day	0	5
7	13.83	Crohn’s disease	Azathioprine 75 mg daily	0	0
8	12.58	Ulcerative colitis	Golimumab 50 mg 4-weekly	25	20
9	13.66	Crohn’s disease	Infliximab 5 mg/kg 8-weekly Azathioprine 100 mg/day	5	0
10	12.33	Crohn’s disease	Infliximab 5 mg/kg 8-weekly	5	0
11	16.33	Crohn’s disease	Azathioprine 75 mg/day	0	0
12	17.75	Ulcerative colitis	Azathioprine 100 mg/day	25	20
13	4.58	Ulcerative colitis	Mesalazine 500 mg/day	0	0
14	23.16	Ulcerative colitis	no treatment at this time	0	0
15	17.33	Crohn’s disease	Infliximab 5 mg/kg 7-weekly	20	15
16	7.58	Ulcerative colitis	Mesalazine 1 g/day Prednisone 20 mg/10 mg/day	40	30
17	15.33	Crohn’s disease	Infliximab 5 mg/kg 8weekly	5	0
18	14.16	Indeterminate colitis	Infliximab 10 mg/kg 6-weekly	0	5
19	3.0	Ulcerative colitis	Mesalazine 500 mg/day Prednisone 15 mg/day	15	10
20	10.66	Ulcerative colitis	Mesalazine 1 g/ day	0	0
21	10.5	Crohn’s disease	no treatment at this point	5	5

### Immunopathologic responses to a single bout of exercise after 8 weeks of physical exercise

Table 2 provides an overview of the descriptive and inferential statistics for increases in slope and area under the curve (AUC) for in ESR and CRP and before and after a single exercise bout (6MWT), before and after 8 weeks of physical exercise. All results have been calculated for slopes and AUCs and are presented separately by time (before vs after intervention), groups (IBD-AD vs. IBD-RE vs. HC), and interaction (group x time).

Over time, the AUC for CRP decreased significantly in IBD-AD and IBD-RE (*p < .*001). By contrast, no significant time effects were observed for ESR slope, ESR AUC and CRP slope. Concerning group effects, no significant results were observed for either slope or AUC in ESR or CRP.

Concerning interactions (time x group), AUC of CRP pre-post comparison was significantly different among the three groups (*p = .*01): While CRP AUC decreased among IBD-AD and IBD-RE, it increased among HC.

## Discussion

The key findings of the present study were as follows: After a single bout of exercise, albumin, hemoglobin, erythrocytes, hematocrit and leukocytes increased in all three groups (IBD-AD, IBD-RE and HC). CRP and thrombocytes were only elevated in IBD-AD and IBD-RE as compared to HC. After 8 weeks of physical exercise, ESR, CRP and thrombocytes significantly decreased in all groups. The intervention did not diminish acute immunopathologic responses after a single bout of exercise.

Following others [24, 25] we expected that a single bout of exercise would increase leukocyte, thrombocyte count and CRP in patients with IBD. Data did confirm this. In IBD-AD and IBD-RE, CRP and thrombocytes were elevated after the single bout of exercise, as compared to HC. Leukocytes, albumin, hemoglobin, erythrocytes and hematocrit were elevated in all three groups after a single bout of exercise. Some previous studies reported no significant exacerbation of subjective or objective disease symptoms [24, 25]. However, Ploeger et al. [24] observed increasing cytokine levels after 4x15s bouts of maximal cycle ergometry. Furthermore, several studies [38, 39] with patients with other chronic diseases corroborated the increased levels of circulating blood markers after a (short) single bout of moderate-to-vigorous physical activity.

While the present data do not allow a deeper introspection into the underlying physiological mechanisms, we speculate, that the increase in circulating blood markers could be caused by decreased plasma volume with an accompanied concentration effect [39], along with an activation of myocytes and macrophages with subsequent upregulation and expression of proinflammatory cytokines, such as CRP [40]. For the leukocytosis, increases in cardiac output and blood flow might have led to increased release of leukocytes from the vascular, pulmonary, hepatic, and/or splenic reservoirs. Equally important are catecholamines and glucocorticoids, which bind and activate exercise-responsive leukocytes during and after exercise. Moreover, exercise increases the HPA axis activity and causes increased release of cortisol, which again affects leukocyte trafficking [41]. Further, hemoglobin is activated in its function as an oxygen carrying pigment of the red blood cells from lungs to all tissue [42]. Similarly, the increased serum albumin levels indicate an increased need to maintain the bodies’ fluid balance and supply proteins needed for growth and tissue repair.

With the second hypothesis we expected that an 8-week physical exercise intervention favorably impacted the inflammatory blood markers among pediatric patients with IBD. As expected [12–14], ESR values, CRP and thrombocytes significantly decreased in all three groups. However, comparing groups, ESR was still highest in IBD-AD, followed by IBD-RE and HC. Concerning interactions, decreased values for ESR, CRP and thrombocytes were observed over time in the IBD-AD and IBD-RE groups, while values increased among HC. However, decreased ESR, CRP and thrombocytes are considered indicators for a diminished inflammation [17, 24]. The current data do not allow a deeper understanding of the underlying physiological mechanisms, and again, we rely on findings of previous studies.

First, physical activity reduces transient stool time and therefore reduces the contact time of pathogens with the gastrointestinal mucus layer and circulatory system [43]. Furthermore, exercise is known to reduce visceral fat mass, subsequently reduces the release of adipokines and introduces an anti-inflammatory environment [44]. Bilski et al. [41] argued that myokines (e.g. IL-6) released during skeletal muscle contraction in exercise inhibited the release of pro-inflammatory mediators. Further, IL-6 stimulated the production of anti-inflammatory factors (IL-1 antagonist and IL-10), inhibited pro-inflammatory cytokine (TNF-alpha) [45, 46] and are related to increases in glucagon-like peptides [7]. In this way, myokines may balance or counteract the effects of proinflammatory stimuli in IBD. Myokines may inhibit the release of proinflammatory mediators from the mesenteric white adipose tissue (mWAT), which is typically deregulated in IBD [43]. The theory of increased cytokines like IL-6 can be connected to our inflammation markers CRP and ESR, since the occurrence of IL-6 strongly correlates with CRP [45].

Second, Chen and Noble [47] hypothesized that exercise induced protective heat shock proteins (HSPS) for the regulation of intestinal inflammation and immunity in animal models. HSPS are known to stabilize denatured proteins, suppress pro-inflammatory transcription factors and decrease the secretion of pro-inflammatory cytokines [47, 48].

With the research question we examined whether the immunopathologic response to a single bout of exercise will be reduced after 8 weeks of physical intervention. Data showed that this was not the case. Longer-term exercise participation did not protect against short-term exercise induced changes in circulating cytokines. However, results confirmed an overall anti-inflammatory effect due to reduction in total inflammation. In summary, the current literature, as well as our findings, point towards an anti-inflammatory effect of exercise, but the exact underlying mechanism remains to be determined.

The strength of the present study is that to the best of our knowledge, it is the first to investigating the effects of a longer-lasting physical exercise intervention in a pediatric group of IBD-patients. Our data supports the hypothesis that exercising has the potential to contribute to inflammation suppression and disease management in pediatric patients with IBD.

On the flip side, several issues warrant against an overgeneralization. First, the sample size was rather small. Second, no non-interventional control group was included; this limited the potential of the study to detect intervention effects. Third, in this study, active vs. non-active IBD was in focus, however, the different etiologies of Crohn’s disease (CD) vs. ulcerative colitis (UC) might involve different mechanisms and should be investigated in the future. Fourth, it is conceivable that the pattern of results might depend on further latent, but unassessed physiological and psychological variables.

## Conclusion

Whereas a single bout of exercise increases albumin, erythrocytes and leukocytes, moderate-intensity aerobic exercise across 8 consecutive weeks reduced inflammatory markers among pediatric patients with IBD. Pediatric patients with IBD should be encouraged to engage in regular moderate-intensity exercise activities, as such activities may contribute to inflammation suppression and improved disease management.

## Data Availability

The datasets used and/or analysed during the current study are available from the corresponding author on reasonable request.

## Abbreviations

6MWT: 6-min walking test
CD: Crohn’s disease
CRP: C-reactive protein
ESR: Erythrocyte sedimentation rate
HC: Healthy control
IBD: Inflammatory bowel disease
IBD-AD: IBD active disease group
IBD-RE: IBD remission group
UC: Ulcerative colitis
